# Assembly of nonheme Mn/Fe active sites in heterodinuclear metalloproteins

**DOI:** 10.1007/s00775-014-1140-7

**Published:** 2014-04-26

**Authors:** Julia J. Griese, Vivek Srinivas, Martin Högbom

**Affiliations:** Arrhenius Laboratories for Natural Sciences A4, Department of Biochemistry and Biophysics, Stockholm Center for Biomembrane Research, Stockholm University, 10691 Stockholm, Sweden

**Keywords:** Affinity, Chlamydia, Cofactor maturation, Metal selection, Specificity

## Abstract

The ferritin superfamily contains several protein groups that share a common fold and metal coordinating ligands. The different groups utilize different dinuclear cofactors to perform a diverse set of reactions. Several groups use an oxygen-activating di-iron cluster, while others use di-manganese or heterodinuclear Mn/Fe cofactors. Given the similar primary ligand preferences of Mn and Fe as well as the similarities between the binding sites, the basis for metal specificity in these systems remains enigmatic. Recent data for the heterodinuclear cluster show that the protein scaffold per se is capable of discriminating between Mn and Fe and can assemble the Mn/Fe center in the absence of any potential assembly machineries or metal chaperones. Here we review the current understanding of the assembly of the heterodinuclear cofactor in the two different protein groups in which it has been identified, ribonucleotide reductase R2c proteins and R2-like ligand-binding oxidases. Interestingly, although the two groups form the same metal cluster they appear to employ partly different mechanisms to assemble it. In addition, it seems that both the thermodynamics of metal binding and the kinetics of oxygen activation play a role in achieving metal specificity.

## Introduction

The metal-based cofactors that are utilized by nature range from the structurally very simple to those with very complex arrangements of metal clusters. In the most basic cases a single metal ion is coordinated by protein side chains, such as in the mononuclear non-heme iron enzymes [1]. Many proteins, however, utilize large and/or multiple metal clusters, such as iron-sulfur cluster proteins [2, 3], the nitrogenase system [4–7], and cytochrome c oxidase [8, 9]. They may also include other non-protein inorganic or organic molecules, such as the hydrogenases [10, 11] or heme [12, 13] and cobalamine proteins [14]. Often metal cofactors, in particular the more complex ones, require elaborate synthesis and assembly machineries. These may consist of numerous proteins that assist in different steps of cofactor synthesis and assembly [15–17]. Identification and characterization of these important machineries is the topic of intense investigation. On the other hand, many cofactors can assemble spontaneously in vitro from the apo (metal-free) protein and metal ions in solution with a varying degree of efficiency. For many of the less complicated cofactors, assembly machineries or specific chaperones have not been identified, and assembly is efficient in vitro [15, 16]. Though this ability does not exclude the possibility that assembly machineries contribute to cofactor formation in vivo [3, 18, 19], it is reasonable to assume that the assembly process for many of these cofactors, also in vivo, is founded in basic chemical principles of metal coordination and binding. In these cases, the protein environment has to provide both affinity and specificity for the correct metal to bind from the complex mixture of the cell.

Many transition metal cofactors are utilized for different types of redox chemistry, commonly following oxygen activation of the cofactor which generates oxidized metal site intermediates [15, 16]. From this perspective, cofactor assembly in these enzymes consists of a two-step process: first, binding of the correct metal ion in its reduced form in the metal-binding site of the protein, and second, the oxygen activation process resulting in the oxidized active cofactor intermediate that ultimately performs the chemistry. As described here, there is evidence suggesting that these processes are not isolated, but can cooperate to achieve correct assembly of the metal cofactor.

## The ferritin-like superfamily and the heterodinuclear Mn/Fe cofactor

The ferritin-like superfamily encompasses several groups of non-heme di-metal carboxylate proteins. In these proteins, the metal ions are coordinated in the center of a 4-helix bundle by four carboxylate and two histidine residues [20–22]. The 4-helix bundle has a particular topology that is shared among the protein groups and, although the sequences have diverged to the point where a common evolutionary origin between many of the groups is not detectable by sequence alone, structural homology and the chemical features of the groups strongly suggest a common ancestry [23]. The protein scaffold binds two metal ions in the +II oxidation state. The metals are then oxidized, commonly by molecular oxygen, producing an oxidized cofactor that is used for performing the chemical function [22–32].

Two of the best-studied groups of the ferritin-like superfamily are the bacterial multicomponent monooxygenases (BMMs) and the ribonucleotide reductase (RNR) R2 proteins. BMMs use an oxidized di-iron cofactor to perform very challenging two-electron oxidations. For example, the soluble methane monooxygenase (sMMO) utilizes an oxo-bridged Fe^IV^/Fe^IV^ intermediate to activate the strongest C–H bond of any saturated hydrocarbon, hydroxylating methane to methanol [31, 33, 34].

Ribonucleotide reductases are the only identified enzyme systems for de novo synthesis of all four deoxyribonucleotides, thus producing the building blocks of DNA [35, 36]. Class I RNR is found in eukaryotes, eubacteria, and a few archaea and consists of two protein subunits, the catalytic R1 subunit and the R2 subunit which generates and provides a radical to the R1 subunit, essential for activity in all RNRs [37]. The R2 proteins are further subdivided into three classes: Ia, Ib, and Ic, depending on allosteric properties and the nature of the metal cofactor and radical species [35, 37, 38]. Chemically, the R2 proteins differ from BMMs in that they perform a one-electron oxidation (radical generation) compared to the two-electron oxidations performed by BMMs. Reduction of molecular oxygen requires four electrons. If these are all taken from the metal ions, both ions are oxidized from the +II to the +IV oxidation state. This Fe^IV^/Fe^IV^ state is the catalytic intermediate of sMMO [34]. In class Ia R2 proteins, on the other hand, an external electron is injected during the oxygen activation reaction, resulting in an Fe^III^/Fe^IV^ oxidation state. This intermediate, denoted intermediate X, oxidizes a nearby tyrosine to a tyrosyl radical which serves as the radical storage site before it is (reversibly) delivered to the substrate-binding R1 subunit for use in catalysis [37, 39, 40]. The class Ib R2 proteins can function with a di-iron site by direct activation of molecular oxygen in a fashion analogous to the class Ia proteins [41, 42]. Interestingly, however, the class Ib proteins also function as di-manganese proteins, but then requiring an additional flavodoxin subunit, NrdI, to provide the oxidant for the metal site. Recent evidence suggests that NrdI utilizes molecular oxygen to produce a superoxide species which is funneled to the reduced di-manganese site and oxidizes it to a III/IV oxidation state that subsequently generates the tyrosyl radical [27, 43–45]. Which cofactor, di-iron or di-manganese, is used chiefly in vivo may differ between species and conditions. There is evidence that the *Bacillus subtilis* and *Corynebacterium ammoniagenes* class Ib R2 proteins are di-manganese enzymes when purified from the native organism, even when overexpressed at non-native levels [46–48] and that the *Escherichia coli* class Ib R2 protein forms a di-manganese site at native expression levels in an Fe-uptake deficient strain grown under Fe-limiting conditions [49].

**Fig. 1:**
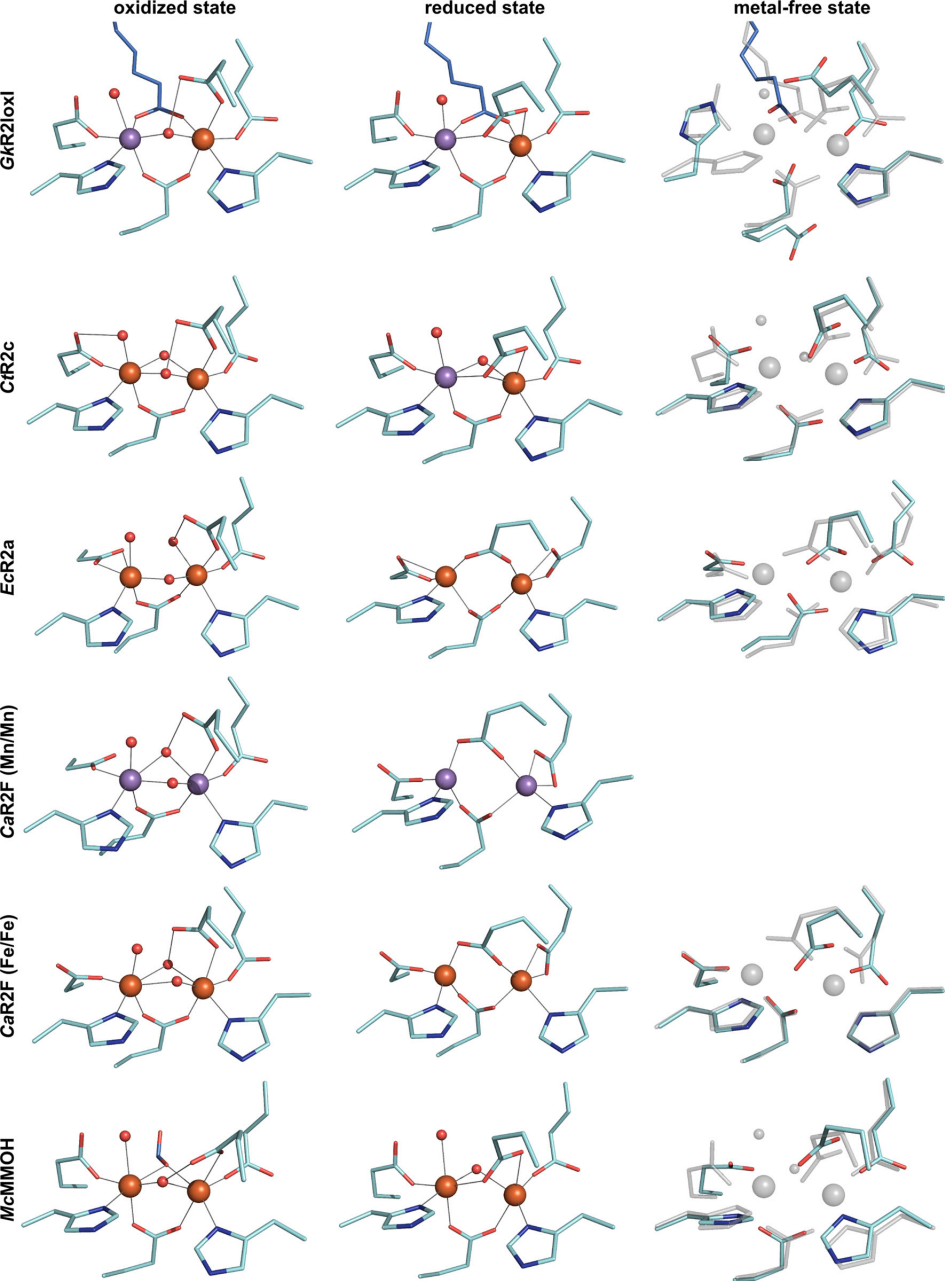
Crystal structures of di-metal-carboxylate proteins with different metal specificities in the oxidized, reduced, and metal-free state. Carbon atoms of metal-coordinating residues are colored cyan, those of exogenous ligands *light blue* (oxygen and nitrogen are *colored red* and *blue*, respectively). Fe and Mn are shown as *orange* and *purple spheres*, respectively, and water/oxo/hydroxo ligands as smaller *red spheres*. Metal–ligand bonds are indicated by *gray lines*. All structures are oriented with site 1 on the *left*, and the metal-free state is always shown superimposed with the reduced metal-bound state in transparent *gray*. *Geobacillus kaustophilus* R2lox homologue 1 (*Gk*R2loxI) binds an exogenous fatty acid ligand. In the oxidized state (4HR0 [28]) it contains a Mn/Fe center, while in the reduced state (4HR4 [28]) site 1 is equally occupied by Mn and Fe (modeled as Mn), and site 2 by Fe. In the metal-free state (4HR5 [28]), site 1 is disordered, whereas site 2 is largely preformed. A structure of the *C. trachomatis* class Ic R2 protein (*Ct*R2c) in the oxidized state is only available with a di-iron cluster (SYY [51]), while in the reduced state exclusive occupancy of a Mn/Fe center was obtained (4M1I [56]). Both sites are ordered in the absence of metal ions (4M1H [56]). The *E. coli* class Ia R2 protein (*Ec*R2a) is active with a di-iron cofactor (oxidized state: 1MXR [124], reduced state: 1XIK [59], metal-free state [125] ). Class Ib R2 proteins likely utilize a di-manganese cofactor in vivo [44, 46–49], but are also functional with a di-iron cofactor [42] and display interesting carboxylate shifts with the different cofactors as well as between species [27]. Shown is the *Corynebacterium ammoniagenes* class Ib R2 protein (*Ca*R2F) in the oxidized (3 MJO [46]) and reduced (1KGP [41]) Mn/Mn and oxidized (1KGN [41]) and reduced (1KGO [41]) Fe/Fe-bound, as well as the metal-free state (3DHZ [126]). *Methylococcus capsulatus* (Bath) methane monooxygenase hydroxylase (*Mc*MMOH) is a representative member of the BMM group of di-metal-carboxylate proteins that utilize a di-iron cofactor (oxidized state with a formate ligand: 1FZ1 [122], reduced state: 1FYZ [122], metal-free state 1XMG [127]). Note that the most N-terminal metal ligand is an aspartate in class Ia and Ib R2 proteins, but a glutamate in class Ic R2, R2lox and BMMs

The discovery of the heterodinuclear Mn/Fe cofactor began with the cloning of the R2 gene from the human pathogen *Chlamydia trachomatis*. The protein showed activity in vitro, despite the fact that the sequence appeared to lack the otherwise essential radical harboring tyrosine residue [50]. The structure of the protein was solved in 2004, confirming the lack of the radical harboring amino acid [51]. Using bioinformatics methods, a number of other R2 proteins that shared this feature were also identified [51]. It was proposed that the tyrosyl radical was replaced by a high-valent form of the metal cluster as the repository for the oxidizing equivalent needed to initiate ribonucleotide reduction, and this new R2 subclass was denoted class Ic [51]. In 2007, it was shown that the activity of the protein was greatly enhanced in the presence of manganese and that the metal site responsible for this activity was a heterodinuclear Mn/Fe cluster [52, 53]. Subsequently, it was demonstrated that the protein group to which the *C. trachomatis* class Ic R2 protein belongs actually consists of two groups of proteins, the R2 proteins and a group of ligand-binding oxidases also forming a heterodinuclear Mn/Fe center, denoted R2-like ligand-binding oxidases (R2lox) [24]. This study also described the structure of the heterodinuclear cluster in the R2lox protein from *Mycobacterium tuberculosis*. The structure revealed that metal binding is specific, with the Mn ion occupying metal position 1 (the N-terminal metal-binding site) and Fe in metal position 2 (Fig. 1). The structure of the Mn/Fe cluster in the *C. trachomatis* class Ic R2 protein (hereafter denoted R2c) was also recently determined, showing the same metal positioning as the *M. tuberculosis* R2lox [54–56]. The two groups of proteins containing the Mn/Fe cluster were further described in a bioinformatics study based on available sequences in the databases [57]. In 2010, around 50 sequences each could be assigned to these two groups. With continuing sequencing efforts these numbers have now grown to over 250 R2lox and 150 R2 sequences lacking the radical harboring tyrosine available in GenBank [58].

## Metal binding and the basis for metal specificity in di-metal carboxylate proteins

The metal-binding sites in most di-metal carboxylate proteins are comprised of two histidines and four carboxylate residues. This coordination sphere is the same for all three types of metal sites, illustrating the challenges in a priori assignment of metal centers based on sequence or structure. In BMMs as well as both groups of Mn/Fe proteins all four carboxylates are glutamate residues. In the class Ia and class Ib R2 proteins, on the other hand, the first carboxylate ligand in the primary sequence is an aspartate (Fig. 1). The ligands bind two metal ions to form a dinuclear metal center that is commonly of a (distorted) octahedral geometry, though there are exceptions [59]. During the oxygen activation reaction, a number of the carboxylate ligands move and change their coordination mode and the number of metal contacts (so-called carboxylate shifts) [31, 59–62].

Mn and Fe are neighbors in the periodic table and can be isoelectronic depending on oxidation state. Their primary ligand and coordination geometry preferences are, therefore, qualitatively similar, thereby complicating direct discrimination between the two metals in a protein binding-site. Nevertheless, there are quantitative differences in their interactions with coordinating ligands that result in differences in the stability of complexes. A convenient way of describing these differences was introduced by Irving and Williams [63]. They found that the stability of complexes of divalent ions of first-row transition metals followed the order Mn^II^ < Fe^II^ < Co^II^ < Ni^II^ < Cu^II^ > Zn^II^, in essence regardless of the nature of the coordinating ligand. This relationship is commonly called the Irving–Williams series. The behavior can be rationalized based on the trends in ionic radii and crystal field stabilization energy of the metals. Cu^II^ represents a special case as it is subject to a strong Jahn–Teller effect (see below) which provides further stabilization of the complex.

The Jahn–Teller theorem in principle predicts that complexes with a degenerate ground state will undergo distortion of the geometry to remove this degeneracy, resulting in a lowering of the energy and stabilization of the complex [64, 65]. For octahedral complexes this usually leads to an elongation of the coordination distances along one of the axes. If the degenerate orbitals point directly toward the ligand, the distortion and stabilization is significantly stronger than otherwise. For high-spin octahedral complexes of Mn and Fe, no Jahn–Teller effect is expected for Fe^III^ and Mn^II^, whereas a strong effect is present for Mn^III^ and, in principle, a weak effect for Fe^II^.

While the Irving–Williams series description is attractive and serves well to describe many observations regarding protein metallation, it cannot easily be applied to biological systems in the cellular context. The concentration of free metal ions in a living cell is very low, and for many metals it is expected to be effectively zero. In vivo, metal ions mainly exist in complex with other ligands, and thus the absolute stability of a complex is less important than the relative stability of the protein–metal complex as compared to the complex between the metal and the original ligands which are released upon metal binding to the protein [15]. Moreover, the behavior predicted by the Irving–Williams series does not help us in understanding the selection of Mn over Fe, as it predicts that Fe would always form a more stable complex than Mn.

The Jahn–Teller effect, on the other hand, could in principle be utilized to tune metal specificity between Mn and Fe. For example, if the metal ions, before binding to the protein, are bound by ligands that adapt to the Jahn–Teller distortion of Fe^II^, while the protein enforces a strict octahedral coordination, it would be expected that Fe^II^ would be less prone to bind there, compared to a more flexible site. This situation would, therefore, in principle increase the Mn^II^ to Fe^II^ ratio in that site. However, the Jahn–Teller effect for Fe^II^ is weak, and metal-coordinating side chains in proteins commonly show a flexibility that would be expected to accommodate these differences. Thus, this effect is unlikely to provide a significant contribution to specificity in practice.

Compared to some of the very complex cofactors mentioned above, assembly of the Mn/Fe cofactor discussed here may appear deceivingly simple at first glance because it consists of just two metal ions coordinated directly by the protein matrix. However, the case appears much more complicated when one considers that the two ions are bound by identical protein ligands, provided in a symmetric fashion by the protein, but nonetheless Mn is found in one site, while the other binds Fe. In addition, the proteins and metal-binding sites that house this heterodinuclear cofactor are very similar to a number of well-studied protein groups that form di-iron centers. Even from high-resolution structures of members of the different families, it is not apparent which proteins will form which particular type of site (Fig. 1). In this case, metal specificity is thus the fundamental problem that must be solved for the cofactor to form correctly. Metal chaperones that specifically deliver metals can be used for these purposes [66–68], in essence transforming a metal-coordination specificity problem to an, arguably simpler, protein–protein interaction specificity problem. Of course, the chaperone itself must also acquire the correct metal by some means. Utilizing different folding compartments with different metal content can also be used to control protein metallation [18, 69]. However, recent studies show that the heterodinuclear Mn/Fe cofactor can be assembled in vitro in the absence of any potential assembly machineries or chaperones [28, 56]. This observation is consistent with the finding that the cofactor can be assembled when proteins are heterologously expressed in *E. coli* [24, 54, 70]. In these cases, the protein scaffold per se is thus able to direct metallation and discriminate between Fe and Mn in the different positions.

## The need for specificity and technical considerations

Metal binding to a protein is ultimately an equilibrium reaction. Although a protein in its native state typically functions with only one type of metal ion, it usually has affinity for other cations in the same chelating position. Thus, if the apo protein is produced and exposed to a sufficient concentration of a different metal, this will generally bind in the same metal-binding site [18, 61, 71–73]. For this reason, exposure of an apo protein to a mixture of metals at different concentrations, as in the living cell, will generally result in a population of proteins with different metals bound. There is usually only one type of metal that provides the activity required for a metalloprotein to be able to fulfill its physiological function. It is thus advantageous for the organism to have a large fraction of the protein population acquire this particular metal, as most other complexes are likely inactive and would be a waste of energy and resources to produce. However, as long as the function can be fulfilled at an acceptable metabolic cost, having subpopulations of mismetallated proteins does not pose a major problem. In some instances, it may even be advantageous for the evolution of new functions or capabilities [18, 30, 73, 74]. The different metal requirements of the different proteins in the ferritin superfamily are likely a result of these evolutionary principles [23].

The conditions in vivo differ from those commonly used to study metalloproteins. Available spectroscopic and crystallographic methods require large amounts of a highly purified and homogeneous sample. Usually, the protein is obtained by heterologous overproduction, a situation that may be very distant from the native environment regarding metal availability, competing proteins, and cofactor assembly machineries [73, 75]. In vitro cofactor assembly protocols are also biased by initial assumptions on what components make up the active metalloprotein. How cofactor assembly is performed in vitro directly influences metallation. For the heterodinuclear cofactor discussed here, sequential addition of metals under different conditions is sometimes required to obtain a large fraction of correctly assembled centers in the sample for further studies. No matter what approaches are taken during in vitro reconstitution or heterologous production, the conditions will, in various ways, differ from those for the assembly of the protein in the native organism at native expression levels. Understanding cofactor assembly and specificity thus necessarily includes piecing together information from different approaches.

Metal ions in a protein sample can be identified and quantified in many different ways, including inductively coupled plasma mass spectrometry (ICP-MS), total reflection X-ray fluorescence (TXRF), proton-induced X-ray emission (microPIXE) [76], and in many cases using specific colorimetric reagents. While these methods allow quantitative determination of the metal content, they do not reveal the location of the metal ions in the protein.

Protein X-ray crystallography is an extremely powerful method that provides a global view of relative atomic positions in a protein and a metal cofactor at atomic resolution [77]. For this reason the method commonly serves as a basis for structural assignment of a cofactor and the mapping of spectroscopic data. Considering the discussion regarding specificity and mismetallation above, however, this method has a general weakness. In a standard crystallographic experiment an electron density map is generated, describing the distribution of electrons in space. This is then interpreted as the protein and any other species bound to it. Atomic assignment of the protein is greatly simplified because we have a priori knowledge of the primary structure, i.e., the amino acid sequence and the chemical structures of the amino acids. Assignment of bound molecules is more difficult, however, especially for monoatomic species like metals. As metals have a large number of electrons and, therefore, result in a relatively large peak of electron density, their position can be accurately determined. The identity of the metal, on the other hand, is more difficult to establish. In practice, it is usually impossible to differentiate between atoms with a similar number of electrons, such as first-row transition metals. In addition, the electron density represents an average of billions of protein molecules in the crystal, and the resulting peak of electron density will reveal no, or very little, information about subpopulations of different metals bound in the same site. This limitation of the method should be considered when assigning crystallographic data and interpreting deposited models in the protein data bank (PDB) [78].

**Fig. 2:**
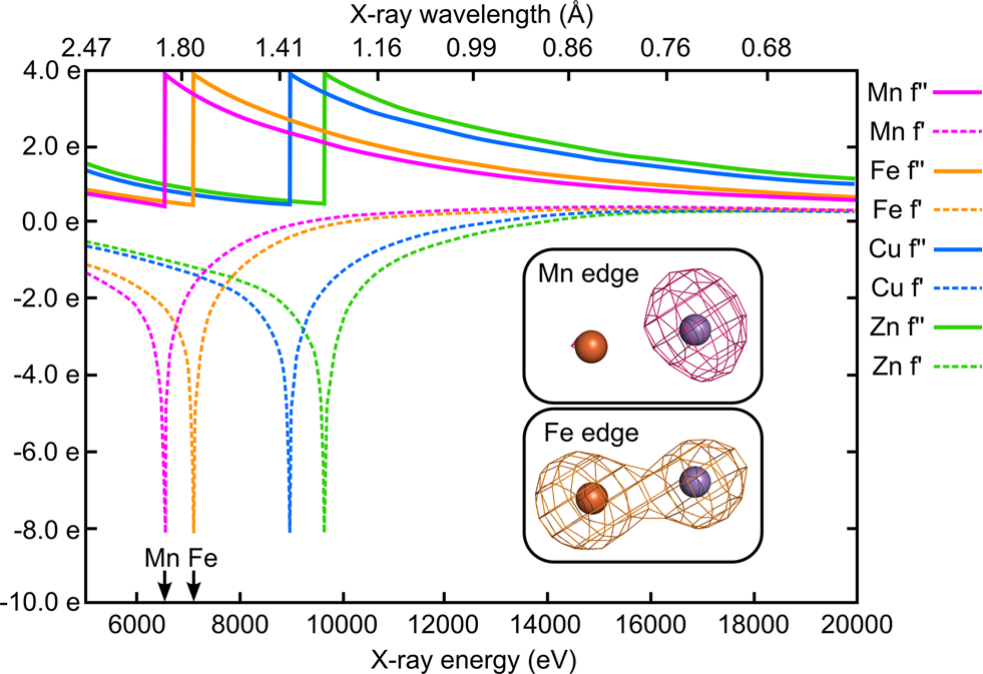
X-ray anomalous scattering. The absorption of X-rays by an element in a crystal has a measurable effect on X-ray diffraction data collected at that element’s absorption edge, termed anomalous scattering or anomalous dispersion. This effect can be visualized in anomalous difference electron density maps. The absorption edges of most elements in proteins are not accessible by synchrotron radiation, but the edges of transition metals which commonly make up metallocofactors are. The graph shows the dispersion, or real (*f′*) and absorption, or imaginary (*f″*) component of the anomalous scattering of X-rays by Mn, Fe, Cu, and Zn. The imaginary component *f″* is proportional to the absorption curve of the element, and the real part *f*′ is mathematically derived from *f″* [77]. The insets show the anomalous difference density from diffraction data collected at the Mn (*pink*) and Fe (*orange*) edges on crystals of metal-free *G. kaustophilus* R2loxI soaked with Mn^II^ and Fe^II^ in the presence of oxygen, contoured at 4 electrons/Å^3^ [28]. At the Mn edge, only Mn displays an anomalous signal, but at the Fe edge there is also considerable signal from Mn. To differentiate between Mn and Fe data therefore have to be collected at both edges, and the Mn contribution subtracted from the Fe contribution [28, 54–56, 79]. Graph adapted from http://skuld.bmsc.washington.edu/scatter/AS_index.html

With X-rays of tunable wavelength, however, advantage can be taken of the fact that atoms absorb X-rays and display absorption edges at specific X-ray energies that are characteristic for each element (Fig. 2). This absorption has a measurable effect on X-ray diffraction data collected at an element’s absorption edge, termed anomalous dispersion or anomalous scattering. From data collected at absorption edges anomalous difference electron density maps can be generated in which not only the location, but also the identity and even relative amounts of different metal ions in a given position can be determined [24, 28, 54–56, 79]. This is a method that has proven central to studying the assembly of the heterodinuclear Mn/Fe cluster.

However, X-ray crystallography alone does not suffice to fully characterize a metalloprotein’s active site. Using anomalous dispersion, we can clearly locate, identify, and even quantify metal ions, but their redox state remains unknown. Moreover, metal centers in crystals can suffer photoreduction in the X-ray beam [41, 59, 80–87]. Crystals are commonly exposed to X-rays at cryogenic temperature where the extent of movement of the metal ions, coordinating side chains, and other ligands upon photoreduction is greatly reduced, but from the structures alone it is usually impossible to tell how much has changed due to photoreduction. Furthermore, short-lived intermediates are often difficult to trap crystallographically. Therefore, other methods are necessary to complete the picture. Most central to the study of metalloproteins are the different spectroscopies that specifically visualize the metal clusters: electron paramagnetic resonance (EPR) [88], X-ray absorption (XAS) [89], and Mössbauer spectroscopy [90]. All these techniques reveal information about the redox state, as well as the electronic environment of the metal ions. EPR detects unpaired electrons in a sample by their absorption of microwave energy in a strong magnetic field. Most elements present in biological molecules contain only paired electrons, with the notable exception of radicals and transition metal ions. Therefore, metal centers can be specifically detected by EPR, given that they are present in a redox state that contains unpaired electrons. Mössbauer spectroscopy is (commonly) limited to samples containing the iron isotope ^57^Fe, so Fe cofactors have to be labeled to be studied. To address the kinetics of metallocofactor-catalyzed reactions, EPR and Mössbauer spectroscopy can be performed in freeze-quench mode. These are very sample-intense techniques, however, and interesting reaction intermediates are, therefore, generally first identified using time-resolved UV/visible or resonance Raman spectroscopy. In addition, computational studies using density functional theory (DFT) models derived from high-resolution structures can greatly aid and extend the interpretation of spectroscopic data.

## Assembly of the Mn/Fe cofactor

To date, three Mn/Fe proteins have been studied experimentally: the *C. trachomatis* R2c as well as the R2lox protein from *M. tuberculosis* and one of the two R2lox proteins (termed homologue 1) from *Geobacillus kaustophilus.* 1 All three proteins were found to bind a heterodinuclear Mn/Fe cluster with the Mn ion in site 1 [24, 28, 52–56]. However, recent work suggests that R2c and R2lox proteins may employ partly different mechanisms to assemble the heterodinuclear cluster [28, 56]. Although caution must be taken in directly comparing the results of these studies because different reconstitution protocols were employed, some differences between the two systems can be identified.

The Mn/Fe cofactor is assembled in at least two steps. In the first step, metal ions in the +II oxidation state bind to the protein active site. Then, oxygen binds and is reduced. In this oxygen activation reaction both metal ions are, at least temporarily, oxidized to the +IV state. In R2c, an external electron is subsequently injected, leading to the active Mn^IV^/Fe^III^ state [52, 91]. The presumed active state of R2lox proteins is the Mn^IV^/Fe^IV^ state. This oxidation state has to date not been directly observed in the R2lox proteins, but its existence is postulated because of the similarity to the R2c system and a number of other observations: oxygen exposure of the Mn^II^/Fe^II^ site in R2lox results in the formation of a crosslink between a tyrosine and a nearby valine, a two-electron oxidation. Computation suggests that the Mn^IV^/Fe^IV^ state is capable of performing the observed chemistry [28]. The reaction with oxygen also results in the resting Mn^III^/Fe^III^ oxidation state of the cofactor, thus, together with the crosslink formation, accounting for all four electrons required for reduction of molecular oxygen.

How the cofactor is assembled and which metal ions are eventually found in the active site may depend on a number of factors. Even if a potential contribution of metallochaperones is excluded, following the equilibrium discussion above, the resulting metal content will be influenced by which metal ions are available in which concentrations, both absolute and relative to each other, how specific each site is for which metal, and what, if anything, happens during oxygen activation. Is metal selection controlled thermodynamically or kinetically, or do both thermodynamic and kinetic effects play a role? Below we summarize the current understanding of these processes.

## Binding of metal ions

R2c and R2lox appear to have different metal preferences in the reduced state. Fe can bind to both metal sites in both systems. Because Fe is much more abundant than Mn in standard *E. coli* growth media [92], R2c was, therefore, first isolated with a di-iron cofactor [51, 93], and it took several years until it became clear that the highest activity was obtained with a 1:1 ratio of Fe^II^ to Mn^II^, suggesting a heterodinuclear cofactor in the protein [52, 53]. R2lox, on the other hand, appears to be less prone to form a homodinuclear Fe/Fe site, as the *M. tuberculosis* protein overexpressed in *E. coli* in rich medium still contained a significant amount of the heterodinuclear cofactor [24]. The fact that Fe readily binds to both sites of R2c and R2lox also presents researchers with considerable difficulty in obtaining homogeneous preparations of the mixed-metal cofactor. From the many different reconstitution protocols that have been tested it is, however, also possible to learn much about the cofactor assembly pathway.

Crystal soaking experiments showed that the two metal-binding sites of R2lox have intrinsically different affinities for either metal. When exposed to a large excess of Mn^II^ and Fe^II^ in equal concentrations in the absence of oxygen, site 2 preferentially binds Fe, as expected based on the Irving–Williams series. In contrast, site 1 binds equal amounts of Mn and Fe [28]. This direct competition experiment has not been performed on R2c, but the site-specific metal content was analyzed following a sequential metal loading scheme. The protein was first exposed to 1 equivalent (per protomer) of Mn^II^, and after introduction into an anaerobic chamber 1 equivalent of Fe^II^ was added. Protein crystals obtained from this mixture contained only Mn in site 1 and only Fe in site 2 [56]. Judging from these results, it appears that site 2 strongly prefers Fe in both R2c and R2lox, whereas site 1 is non-specific in R2lox, but prefers Mn over Fe in R2c.

Other experiments shed some more light on the different metal-binding behavior of the two systems. As discussed above, if only one type of metal ion is available in very high concentrations it is likely to bind to both metal sites [18, 41, 61, 73]. However, such experiments are very distant from metal-binding conditions in vivo. If R2c is incubated with substoichiometric amounts of Mn only, Mn binds in both or either of the two metal-binding sites of R2c [56]. (From these crystallographic experiments it is impossible to tell whether the observed anomalous difference density for Mn in both sites stems from simultaneous or alternate occupation of the two sites, or a mixture of these in the crystal.) Exposure to excess Mn during cocrystallization or crystal soaking leads to a maximum occupancy of 90 % Mn in site 2 and 70 % in site 1 [56]. In contrast, Mn does not bind in any significant amount in site 2 of R2lox. In fact, on its own, Mn only binds weakly and transiently even in site 1 of R2lox [28]. It should be noted, however, that the different reconstitution protocols employed might have influenced the results obtained: Mn was observed in both sites of R2c after cocrystallization or crystal soaking with Mn [56]. Mn-only samples of R2lox were prepared using a fourfold molar excess of Mn over polypeptide chains, and since these samples were prepared for EPR analysis, the preparation included a purification step to remove excess Mn^II^ [28]. This step could also remove labile bound Mn^II^ from the protein. It cannot be excluded that this procedure would have given a similar result for R2c.

Nevertheless, the protocols used to reconstitute the activated Mn/Fe cofactor in R2c and R2lox indicate that the two metal-binding sites do exhibit different preferences for Mn in the two systems. Mn has not been observed in site 2 of R2lox when reconstituted at a protein:Mn:Fe ratio of 1:2:1, whereas both Mn/Fe and Fe/Mn centers (but not Mn/Mn centers) are formed in R2c at similar protein:Mn:Fe ratios [28, 55]. Several slightly different protocols have been used to reconstitute the Mn^IV^/Fe^III^ cofactor in R2c, but in most of them a slight excess of Mn is added to the protein first, followed by addition of substoichiometric amounts of Fe in the presence of oxygen. These procedures typically yield 90 % mixed-metal and 10 % di-iron cofactors [55, 94]. In contrast, when Mn is added first to R2lox, only ~5 % of Mn/Fe cofactors are obtained (our unpublished data).

It is interesting to note that the observed differences in metal specificity between R2c and R2lox also relate to structural differences: site 2 is preformed in the apo protein in both R2c and R2lox, and no major conformational rearrangement is required to accommodate the Fe ion [28, 56]. In contrast, site 1 behaves differently in the two systems. While in R2c site 1 is also ordered in the metal-free state [56], in R2lox it is disordered [28]. Together with the observations from different reconstitution schemes, this indicates that in R2lox Fe has to bind first in site 2, followed by binding of Mn or Fe in site 1, suggesting that metal binding is cooperative. In contrast, metal binding in R2c was proposed to be non-cooperative [56], but hard evidence for either suggestion is not available at this point.

**Fig. 3:**
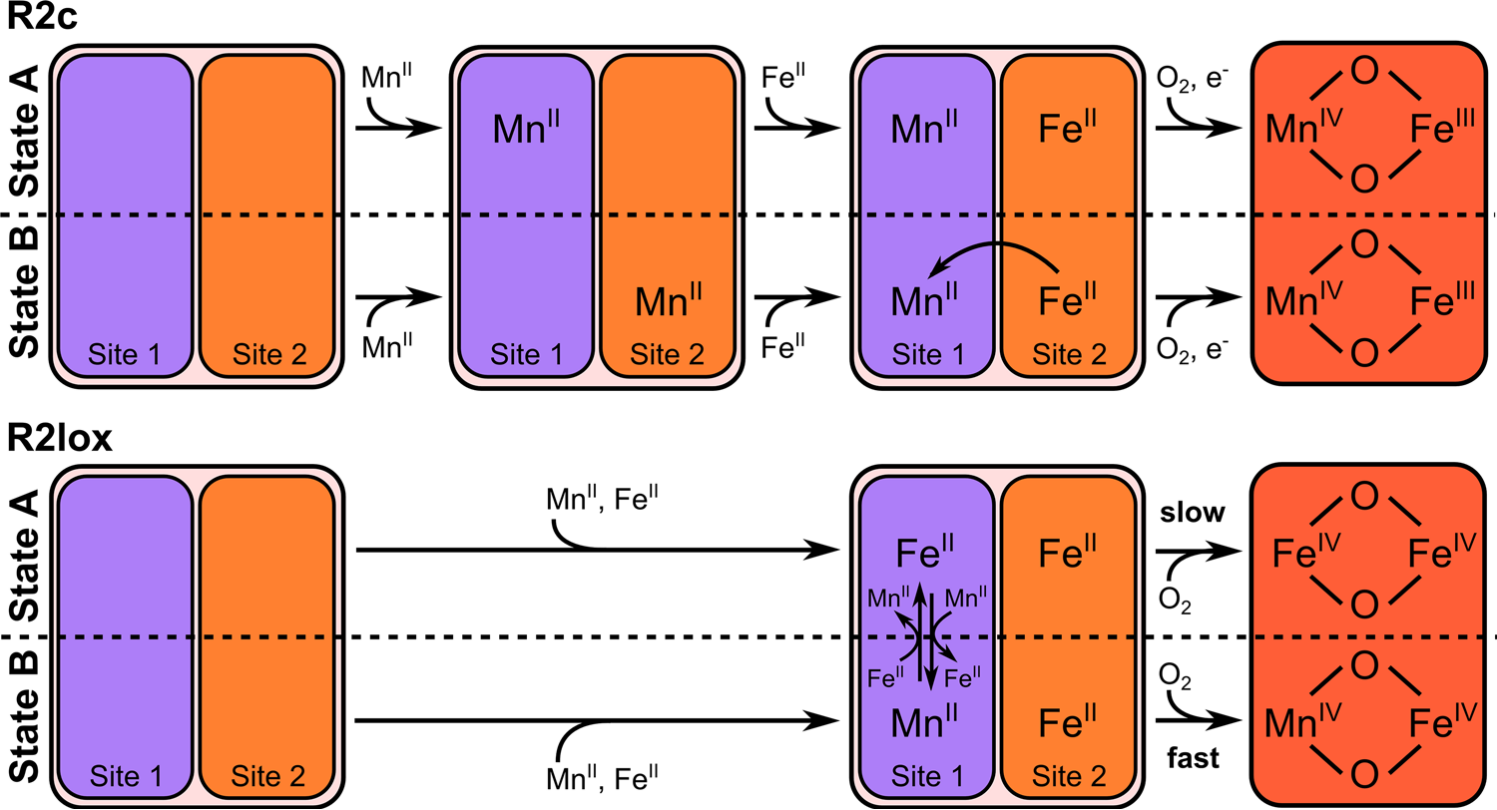
Models for cofactor assembly and maturation in R2c and R2lox proteins. The model for R2c is derived from a sequential loading scheme [56]. When the protein is incubated with Mn^II^ alone, Mn^II^ will bind in either site 1 or site 2 with roughly equal probability. Subsequent addition of Fe^II^ in the absence of oxygen leads to Fe^II^ binding in site 2, thereby displacing any Mn^II^ ions bound in site 2 to site 1 (State B). The heterodinuclear cluster thus assembled then reacts with oxygen and forms the catalytically active Mn^IV^/Fe^III^ state upon concomitant injection of an external electron. In R2lox, when exposed to equal concentrations of Mn^II^ and Fe^II^ simultaneously, site 2 is filled with Fe^II^, and site 1 has approximately equal probability of binding either a Mn^II^ or Fe^II^ ion [28]. The protein-bound metal ion is in equilibrium with solvated metal ions in solution. Subsequent reaction with O_2_ generates the putative catalytically active IV/IV state. The exchange of the reduced ion in site 1 with ions in solution, combined with a significantly faster reaction of the heterodinuclear (Mn/Fe) center with O_2_ compared to that of the homodinuclear (Fe/Fe) center [96], results in accumulation of the oxidized Mn/Fe cofactor over the Fe/Fe cofactor. Note that this model predicts cofactor identity to be influenced by the concentrations of Mn^II^ and Fe^II^ in solution due to their impact on the kinetics of metal exchange

If only Mn is present, it binds in site 1 of R2lox and appears to thereby inhibit binding of Fe added later (but is then lost during purification). In R2c, on the other hand, Mn binds in both sites or either site, and when Fe is then added later (in the absence of oxygen), Mn/Fe cofactors are formed with high efficiency. A possible interpretation of these data is that in R2c the main driving force for formation of the Mn/Fe cofactor is the preference of Fe for site 2, rather than a preference of Mn for site 1 [56] (Fig. 3). If this is the case, the Mn/Fe cofactor in R2c will only be efficiently assembled if Fe is substoichiometric, and if oxygen is excluded until equilibrium has been reached, as both Mn/Fe and Fe/Fe centers (but not Mn/Mn centers) react with oxygen and are thereby “trapped” [44, 95, 96].

These models are derived from different experimental procedures, making direct comparisons difficult. Nevertheless, currently available data do allow the conclusion that metal binding proceeds via different pathways in R2c and R2lox, although the presently proposed models undoubtedly will be refined in the future.

## Cofactor activation

As a background to oxygen activation by the heterodinuclear Mn/Fe cofactor, it is of use to first briefly describe the reaction and intermediates in the extensively studied di-iron cofactor of the class Ia RNR R2 protein (R2a).

**Fig. 4:**
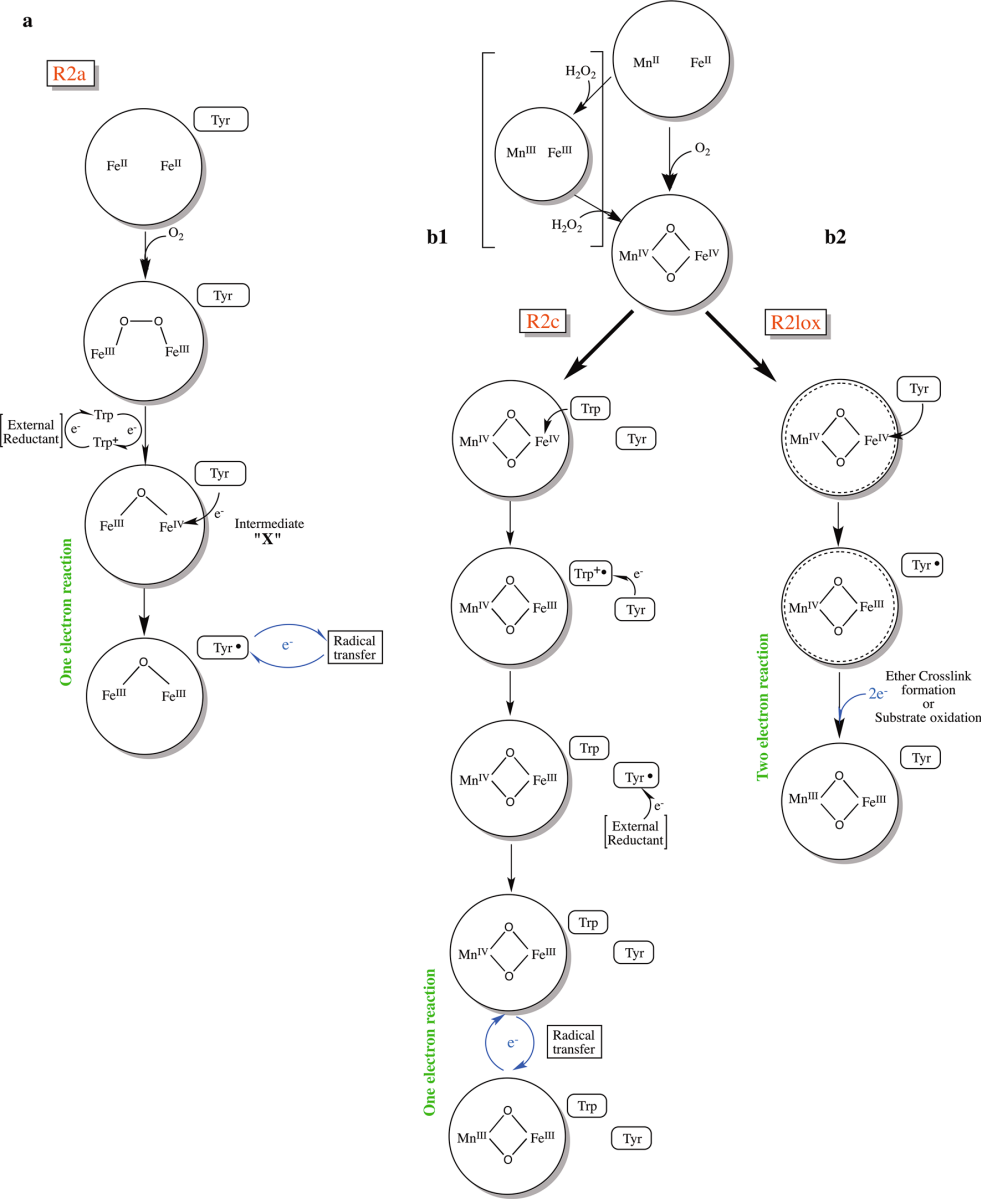
Comparison of the oxygen activation and reaction mechanism of **a** R2a, **b1** R2c and **b2** R2lox. Transition states of metal cofactors encircled in a *dotted* pattern are based on computational studies and have not been experimentally observed

In R2a, reduction and cleavage of molecular oxygen eventually lead to formation of a stable tyrosyl radical (Y·) (Fig. 4). The reaction can be formally written as

$${\text{R2}} {-}{\text{Tyr}} {-} {\text{OH}} + ({\text{Fe}}^{ 2+ } {\text{Fe}}^{ 2+ } ) + {\text{O}}_{ 2} + {\text{H}}^{ + } + {\text{e}}^{ - } \to {\text{R2}} {-} {\text{Tyr}} {-} {\text{O}}^{\cdot} + ({\text{Fe}}^{ 3+ } {-} {\text{O}}^{ 2- } {-} {\text{Fe}}^{ 3+ } ) + {\text{H}}_{ 2} {\text{O}}$$

The radical that resides on the tyrosyl residue in the R2 subunit (Y122 in *E. coli* R2a) is reversibly transferred via a defined radical transfer/translocation/hole translocation pathway to a conserved cysteine residue in the active site of the RNR R1 subunit. The resulting cysteinyl radical then initiates the radical-based substrate reduction mechanism [37, 38, 97, 98].

 Oxygen activation by the reduced, carboxylate bridged Fe_2_^II/II^ metal center in R2a proteins proceeds via a di-ferric peroxo intermediate, observed in *E. coli* R2a mutants, and also in the wild-type R2a from mouse [99–102]. The complete reduction of molecular oxygen results in the key intermediate “X”, directly preceding the tyrosyl radical. At this stage the metal site is in the Fe^III^/Fe^IV^ state. The fourth electron required for complete oxygen reduction is initially supplied by a near surface tryptophan residue (W48 in *E. coli* R2a) that is transiently oxidized to a tryptophan cation radical [38, 98, 103, 104]. The tryptophan radical is rapidly reduced by an external reductant. Intermediate X goes on to oxidize the nearby tyrosine, forming the active state of the protein with a µ-oxo-bridged diferric site and a tyrosyl radical [32, 39]. Intermediate X has been extensively studied spectroscopically and computationally. Mössbauer and ^57^Fe-ENDOR experiments show a *S*_Total_ = 1/2 ground state as the net result of antiferromagnetic coupling between Fe^III^ (*S* = 5/2) and Fe^IV^ (*S* = 2) at the metal center [105]. Structurally it is characterized as a bis-µ-oxo-Fe_2_^III/IV^ or µ-oxo-µ-hydroxo-Fe_2_^III/IV^ site with a coordinated hydroxide, although the details of the structure are still under debate [39, 60, 105–112].

In class Ic, on the other hand, the reduced Mn^II^/Fe^II^ metal center initially provides all four electrons required for complete O_2_ reduction, resulting in a Mn^IV^/Fe^IV^ intermediate [113]. This intermediate, detected by a combination of EPR and Mössbauer spectroscopy, shows a *S*_Total_ = 1/2 ground state resulting from antiferromagnetic coupling between Mn^IV^ (*S* = 3/2) and Fe^IV^ (*S* = 2) at the metal center [113]. The Mn^IV^/Fe^IV^ intermediate has been suggested to have a bis-µ-oxo diamond core structure, in analogy to the Fe^IV^/Fe^IV^ intermediate Q found in sMMO [34, 114]. The Mn^IV^/Fe^IV^ intermediate is formed first-order based on oxygen concentration and no potential preceding intermediates, such as the peroxo species in R2a proteins, accumulate during the reaction [113, 114]. Oxygen is thought to first add to the metal ion in site 2 in all three classes of R2 (Fig. 1) [27, 46, 56, 60, 62, 115, 116]. Because an analogous peroxo intermediate to R2a has not been observed in R2c, it was proposed that addition of O_2_ to the Fe ion might yield a Mn^II^/Fe^III^-η^2^-superoxo complex from which the Mn^II^ could attack the dioxygen bond and immediately generate the observed Mn^IV^/Fe^IV^ intermediate [56]. This should be possible due to the unusually short 3.2 Å distance between the two metal ions in reduced R2c [56]. It should be noted that the metal ions in reduced R2lox are at a distance of 3.6 Å [28]. However, this structure represents a mixed occupation of Mn and Fe in site 1, and we cannot rule out that a shorter distance would be observed if site 1 was exclusively occupied by Mn. Conversely, theoretical modeling of the oxygen activation reaction suggests that molecular oxygen is cleaved by the reduced Mn/Fe metal center, forming a diamond-shaped Mn^III^/Fe^III^ peroxo complex. Mn^III^ is the only redox active species in the transition state and is oxidized to Mn^IV^, leading to the homolytic cleavage of the dioxygen bond in a symmetric diamond-shaped transition state. This is followed by immediate transfer of an electron from the Fe^III^ ion to oxygen, resulting in the Mn^IV^/Fe^IV^ state [96].

**Fig. 5:**
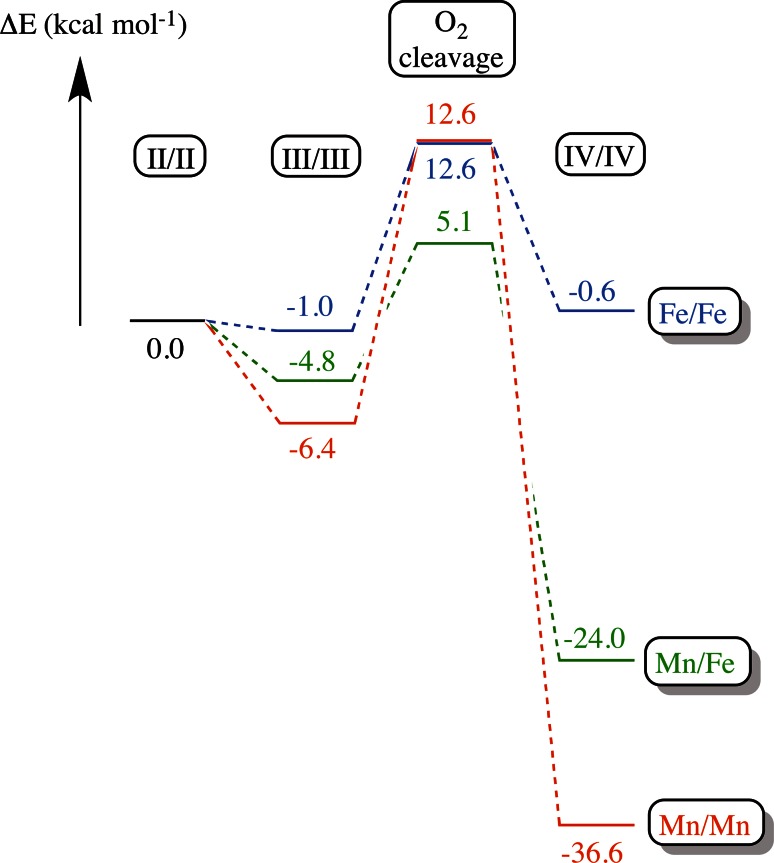
Calculated energy profiles of oxygen cleavage in R2c with different combinations of metals, adapted from [96]

The modeling also suggests that the barrier for oxygen cleavage by the Mn/Fe cofactor is some 3–4 kcal mol^−1^ lower than for the Fe/Fe cofactor, resulting from the higher stability of Mn^IV^ compared to Fe^IV^ (Fig. 5) [96]. This would imply that oxygen cleavage is significantly faster with the heterodinuclear cofactor. Notably, these calculated barriers are for formation of the IV/IV state. The calculations do not take injection of an external electron into account, which occurs in R2c (but presumably not in R2lox, see below) and would influence the experimentally observed rates. Oxygen cleavage by an Mn/Mn center is predicted to have a very high barrier, mainly due to the stability of the peroxo species (Fig. 5) [96]. The calculated energy profiles suggest that Mn^III^/Fe^III^-peroxo and Mn^III^/Mn^III^-peroxo species can be formed; however, to date no peroxo species has been experimentally observed in R2c or R2lox.

Following complete O_2_ reduction the Mn^IV^/Fe^IV^ metal center in R2c undergoes a one-electron reduction resulting in the Mn^IV^/Fe^III^ active state of the protein. Similar to the injection of the “extra” electron during oxygen activation in R2a, this reaction is proposed to proceed via W51, the residue equivalent to W48 in *E. coli* R2a. Y222 in R2c, a residue conserved among the R2c, but not R2lox proteins, has also been shown to mediate this reaction [57, 113]. Together the data suggest a two-step mechanism via Y222 and W51 for the one-electron reduction of the Mn^IV^/Fe^IV^ intermediate in R2c.

The active Mn^IV^/Fe^III^ state has been characterized as an antiferromagnetically coupled Mn^IV^ (*S* = 3/2)–Fe^III^ (*S* = 5/2) cofactor, resulting in a *S* = 1 (EPR silent) ground state [52, 117]. It was also recently studied using a combination of nuclear resonance vibrational spectroscopy, absorption/circular dichroism/magnetic CD/variable temperature, variable field MCD spectroscopies, along with time-dependent DFT [118]. The results suggest the active cofactor to be a μ-oxo, μ-hydroxo-bridged metal center with a terminal hydroxo ligand residing on the Mn^IV^. The hydroxo ligand provides a high proton affinity site on Mn^IV^, suggested to aid radical transfer between the R2 and R1 subunits.

R2c utilizes the Mn^IV^/Fe^III^ oxidation state in place of the tyrosyl radical to activate the RNR R1 catalytic subunit [52, 91, 113]. Calculations suggest that the radical equivalent Mn^IV^/Fe^III^ redox state in R2c is an equally strong oxidant as the tyrosyl radical in *E. coli* R2a [119], so the redox potential of the Mn^IV^/Fe^III^ state is likely commensurate with that of the active site cysteinyl radical in the R1 subunit. The heterodinuclear cofactor may thus represent a pure bioinorganic solution to produce a metal-centered radical-equivalent state allowing reversible radical transfer which the Fe^IV^/Fe^III^ center would probably not allow as it would be too strong an oxidant.

**Fig. 6:**
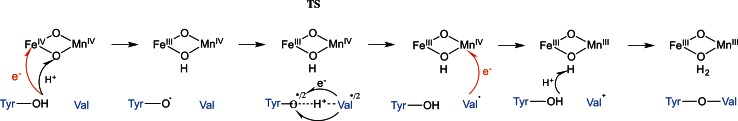
The proposed reaction mechanism for the tyrosine–valine ether crosslink formation observed in R2lox, based on theoretical modeling, adapted from [28]

R2lox, unlike R2a and R2c, performs a two-electron oxidation reaction, similar to BMMs. sMMO, for example, uses an oxygen activated di-iron metal center to perform the two-electron hydroxylation of methane [31, 34]. R2lox is capable of using the Mn/Fe center for the same type of reaction: the Mn^II^/Fe^II^ site catalyzes formation of an unprecedented tyrosine-valine ether crosslink upon oxygen activation [24, 28]. This reaction is formally a 2-electron oxidation with the removal of 2 protons. Theoretical modeling of this reaction suggests that the Mn^IV^/Fe^IV^ state, generated by oxygen cleavage analogous to R2c, oxidizes Y162 to a tyrosyl radical, reducing Fe^IV^ to Fe^III^. This radical is then transferred to V72, creating a valyl radical intermediate. A second electron is subsequently transferred to Mn^IV^ from V72, producing the tertiary valine carbocation V72^+^. This is followed by a nucleophilic attack of the Y162 phenolic oxygen on the Cβ of V72^+^, thereby forming the crosslink and leaving the cofactor in the observed Mn^III^/Fe^III^ state (Fig. 6) [28]. Using time-resolved spectroscopic studies it will be possible to probe this proposed mechanism experimentally.

The physiological function of R2lox proteins is to date unknown. Modeling of the crosslink formation reaction suggests that from the valine carbocation state, desaturation of the valine is also possible. Therefore, it was proposed that R2lox may function as a desaturase, performing tyrosyl radical mediated two-electron desaturations of bound substrates [28]. R2lox proteins have a conserved tyrosine that lines the ligand-binding cavity [57]. This residue (Y175 in *G. kaustophilus* R2lox homologue 1) is positioned at a similar (~5 Å) distance from the metal site as Y162, as well as the radical harboring tyrosine in class Ia R2 proteins, and may be involved in a substrate oxidation reaction, analogous to Y162 in the crosslink formation. Theoretical comparison of potential two-electron chemistry performed by the Mn/Fe heterodimer and the Fe homodimer suggests that the redox potential of the Mn^IV^/Fe^IV^ site is about 7 kcal mol^−1^ lower than that of an Fe^IV^/Fe^IV^ site. While this is likely not enough to oxidize methane to methanol, it suggests that the Mn^IV^/Fe^IV^ site can function as an oxidase for larger exergonically bound substrates [120].

## Cofactor maturation

As detailed above, when crystals of R2lox are soaked with a large excess of Mn^II^ and Fe^II^ in equal concentrations in the absence of oxygen, site 1 is non-specific, binding equal amounts of Mn and Fe, while site 2 binds mainly Fe. Interestingly, however, in the presence of oxygen only Mn is observed in site 1, while site 2 still contains mostly Fe [28]. Based on computational data indicating that the oxygen activation reaction (formation of the IV/IV state) is significantly faster with the Mn/Fe than with the Fe/Fe cofactor [96], we proposed a model for enrichment of the Mn/Fe cluster through oxygen activation (Fig. 3). Metal ions are labile bound in the two sites as long as the cofactor remains reduced and can, therefore, likely exchange until oxygen activation “fixes” the oxidized metal ion complex in the binding site [95]. If metal exchange is fast compared to oxygen activation, oxygen activation will preferentially “trap” the heterodinuclear cofactor over the di-iron center. This model predicts that if metal ions are added in low or no excess over binding sites, the Mn/Fe cluster will not be significantly enriched, and the same percentage of Mn/Fe centers will be obtained in the absence or presence of oxygen. This was indeed found to be the case in EPR samples of R2lox reconstituted in this manner [28]. While the model remains to be thoroughly tested, these data indicate that cofactor assembly in R2lox is controlled thermodynamically, through metal-binding preferences, and kinetically, through reactive differences between the different metals. The efficiency of formation of the mixed-metal cluster, therefore, depends not only on the accessibility of the metal ions, but also on the rate of metal exchange relative to the rate of oxygen binding, suggesting that cofactor assembly in vivo might be regulated by controlled delivery of either metal ions or oxygen to the protein.

**Fig. 7:**
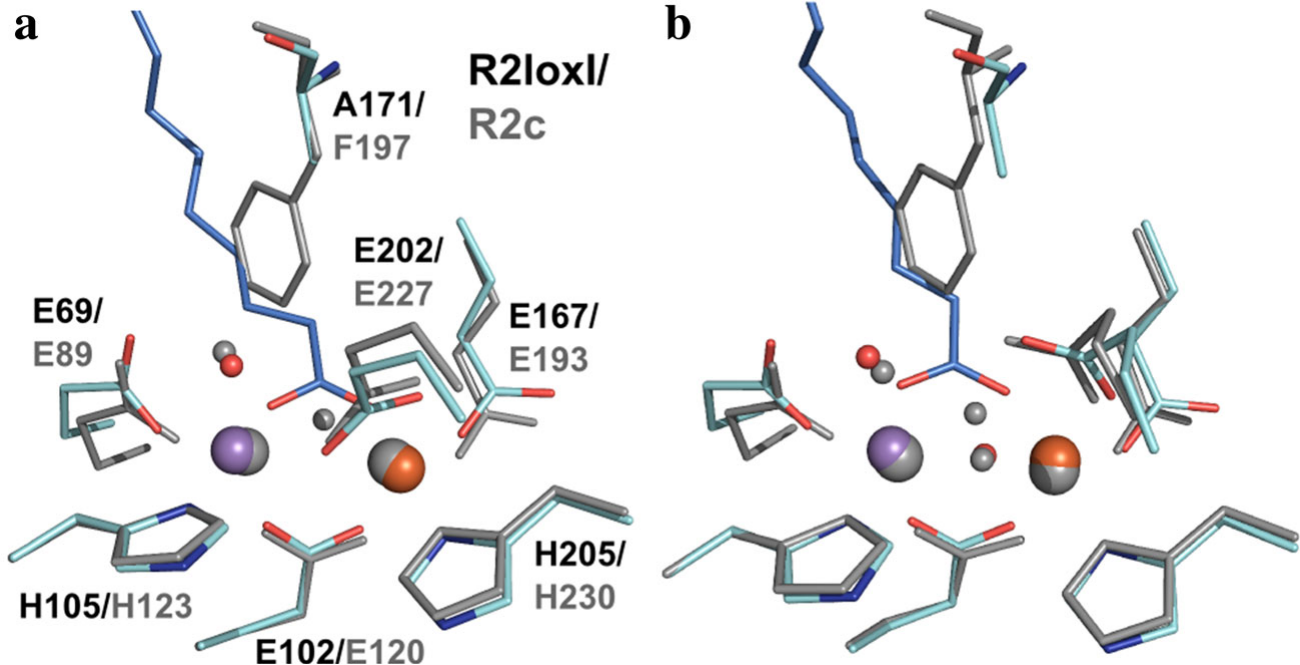
Structural comparison of R2c and R2lox. **a** Superposition of the reduced Mn/Fe-bound states of R2lox (4HR4 [28]) and R2c (4M1I [56]). **b** Superposition of the oxidized Mn/Fe-bound state of R2lox (4HR0 [28]) and the oxidized Fe/Fe-bound state of R2c (SYY [51]). Amino acid residues of R2lox are shown in *cyan*, the fatty acid ligand in *blue*, and Mn and Fe as *purple* and *orange spheres*, respectively, while R2c is shown in *gray*

Such cofactor maturation through oxygen activation has not been described for R2c, but would also not be necessary if, as proposed, cofactor assembly is entirely thermodynamically controlled by the apo protein structure [56]. It should be noted that this thermodynamically controlled assembly mechanism can only function if equilibrium is reached before oxygen activation, i.e., if kinetic effects are excluded. The effect of oxygen on metal loading/specificity in R2c has not been thoroughly investigated to date. However, using aerobic sequential loading schemes, a significant percentage of Fe/Fe cofactors was formed even if Fe was added substoichiometrically [55, 94]. A direct competition experiment analogous to that performed with R2lox would be very informative to further elucidate the mechanism of cofactor assembly in R2c. It is at this point not possible to exclude that similar results as for R2lox would be obtained, although it would be expected that both the rate of oxygen activation (see above) and the rate of metal exchange in R2c and R2lox differ. R2c has two preformed metal coordination positions, while in R2lox site 1 is structurally dynamic and becomes ordered only upon metal binding. Moreover, whereas in R2c the active site is buried deep within the protein and isolated from solvent [51, 56], R2lox proteins have a large ligand-binding channel leading from the protein surface to the active site [24, 28]. In the outer ligand sphere, residue F197 of R2c is positioned between the two metal sites and was proposed to prevent cooperativity between them [51]. In R2lox this residue is replaced by an alanine (A171). Several such substitutions of large for small side chains generate the ligand-binding channel in R2lox [24, 57]. A phenylalanine in place of A171 would indeed block ligand binding (Fig. 7). Therefore, the metal-binding sites are more accessible in R2lox than in R2c. It appears likely that the exchange rate for metals bound in R2c is lower than in R2lox, while oxygen activation is faster due to injection of an external electron, and the heterodinuclear cluster would not be significantly enriched in R2c upon oxygen activation. Hence, differences in the cofactor assembly mechanisms might have evolved along with, or even because of, the structural and functional changes of these two enzyme systems.

In summary, currently available data suggest that in R2c, both Mn and Fe can bind in both sites, and a preference of Fe^II^ for site 2 drives formation of the Mn^II^/Fe^II^ cluster rather than the Fe^II^/Mn^II^ cluster provided that Fe is substoichiometric. In R2lox, on the other hand, it appears that Mn preferentially binds in site 1, while Fe can bind in both sites, and metal exchange in site 1 coupled with a faster oxygen activation rate of the heterodinuclear cluster drives formation of the oxidized Mn/Fe rather than the Fe/Fe center (Fig. 3). In other words, in R2c Fe is enriched in site 2 through thermodynamic effects, while in R2lox Mn is enriched at site 1 through kinetic effects, in both cases leading to formation of a heterodinuclear Mn/Fe cluster with the Mn ion in site 1.

## The structural basis for metal specificity

With more and more high-resolution structures of R2 and R2-like proteins becoming available, it is growing increasingly clear that there is no simple explanation for the differential metal preferences these proteins display. One would like to be able to point at a certain amino acid residue or combination of residues as the basis for specificity, but the situation appears to be much more complicated. Notably, the tempting conclusion that the identity of the N-terminal metal ligand is responsible for the metal specificity of site 1 does not hold up. This residue is an aspartate in the di-iron class Ia and di-iron or di-manganese class Ib R2 proteins, but a glutamate in the Mn/Fe proteins, as well as the di-iron-binding BMMs (Fig. 1). As mentioned above, there is large structural flexibility in the metal-binding sites, and some proteins are observed having different metal ions bound, but with nearly identical active site geometries (Fig. 1). Nevertheless, certain differences which may be meaningful can be observed.

The geometries of the primary coordination spheres are nearly identical in R2c and R2lox, both in the reduced and the oxidized state (Fig. 7) [24, 28, 51, 56]. It should be noted, however, that the oxidized state structure of R2c was obtained with a di-iron cofactor. Structures of the oxidized Mn/Fe-bound state of R2c have to date only been obtained with mixed or substoichiometric metal loading and are inconclusive [54, 55]. The most marked difference between R2c and R2lox is that in R2lox a fatty acid ligand bridges the metal ions in place of the hydroxo bridge of R2c. The oxygen-derived bridging ligand present in the oxidized state is an oxo anion in R2c, whereas it is a hydroxo anion in R2lox [28, 51, 56, 118]. It must be presumed that the particular copurifying fatty acid ligand in R2lox is an artifact from heterologous overproduction, a compound which likely bears similarity to a natural substrate, but is not turned over and released from the protein. During a normal oxygen activation reaction, an exogenous substrate will likely not directly coordinate the metal ions at all times, as it would require that protein-derived metal ligands are displaced to allow for oxygen binding [28, 96]. The reaction intermediates might therefore display very similar active site configurations as in R2c.

In both systems, both sites have distorted octahedral coordination spheres, with the distortion being more pronounced at site 2. Mn^II^ prefers perfect octahedral coordination geometries, whereas Fe^II^ displays a minor Jahn–Teller effect, so that the geometry of site 2 is perhaps more favorable towards Fe binding. The comparison with other di-metal-carboxylate proteins reveals that both metal-binding sites generally have distorted coordination spheres, and the distortion is generally stronger in site 2. Interestingly though, the iron-binding sites tend to be more distorted than manganese-binding sites (Fig. 1).

Another notable difference between the two sites in the Mn/Fe proteins is that one of the metal ligands in site 1 is water, perhaps lending more geometrical flexibility to this site compared to site 2, which has only protein ligands in the reduced state. This greater flexibility might allow site 1 to accommodate both Mn and Fe, while site 2 prefers Fe. However, BMMs also have a water ligand in site 1 (Fig. 1) [121, 122], yet are found to function with a di-iron cofactor [123]. In short, as stated at the beginning of this section, the case is clearly quite complicated, and we are yet far from resolving how structure directs metal specificity, although we can at this point firmly say that it does.

## Conclusions

In R2c the Mn/Fe cofactor is assembled efficiently only if Fe is present in substoichiometric amounts. This proposal is in line with the speculation that the Mn/Fe cofactor may be an adaptation to iron limiting conditions [56]. However, R2c is only highly active with a mixed-metal cofactor [52, 53], and, therefore, organisms containing only a class Ic RNR would have to always live under iron-limiting conditions to have an efficiently assembled R2c subunit. We know too little about the general cellular metal status of most organisms to be able to say whether this is indeed the case. This also relates to the question why the heterodinuclear cofactor evolved. One hypothesis regarding its use in R2c is that the active Mn^IV^/Fe^III^ state is less sensitive than the tyrosyl radical to some radical scavengers such as nitric oxide produced by the immune system of the host [51]. This would be consistent with the observation that these enzymes are primarily found among extremophiles and pathogens that reside in particularly hostile environments [57]. It also finds support in that the active state in R2c is stable to incubation with hydrogen peroxide and that the active Mn^IV^/Fe^III^ state is even efficiently assembled by incubation of reduced forms of the protein with hydrogen peroxide [94] (Fig. 4).

To date it has not been shown that any R2c or R2lox protein indeed binds a Mn/Fe cluster in vivo. Given available data, however, this seems likely. In the case of R2c the protein is only highly active with the heterodinuclear cofactor [52, 53]. For R2lox the case is less clear cut, at least as long as its in vivo activity remains unknown. The di-iron cofactor of BMMs catalyzes two-electron oxidations from the IV/IV state, and hence it appears likely that R2lox may function with both a Mn/Fe or a Fe/Fe cofactor. The rationale for employing the more complex heterodinuclear cofactor in this case could then be the greater stability of its high valent state [120]. It is also possible that the oxidation potential of the Mn^IV^/Fe^IV^ state in R2lox is commensurate with the intended substrate, thus reducing the risk of detrimental side reactions that may occur with the more reactive Fe^IV^/Fe^IV^ cofactor. This reasoning is thus partly analogous to the balancing of the Mn^IV^/Fe^III^ state in R2c with the active site cysteinyl radical in the R1 subunit.

Alternatively, R2lox might utilize either a Mn/Fe or a di-iron cofactor depending on the cellular metal status. In contrast, R2c has to be very selective because there is no tolerance for any other than the Mn/Fe cofactor. We, therefore, speculate that the different chemistry the two systems perform may reflect on their cofactor assembly mechanisms.

As discussed above, the cellular environment influences metal loading of proteins by providing the basis for metal availability, alternate chelating groups, and potential metal chaperones. However, available data show that the R2c and R2lox proteins exhibit intrinsic metal specificity in vitro, allowing them to discriminate between Mn and Fe and promote formation of the correctly assembled heterodinuclear cofactor. Although a definitive structural or chemical answer as to how is still elusive, it is interesting to note that it appears that both the thermodynamics of metal binding and the kinetics of oxygen activation likely play a role in reaching this specificity.

## Abbreviations

BMM: Bacterial multicomponent monooxygenase
DFT: Density functional theory
EPR: Electron paramagnetic resonance
R2a: Class Ia ribonucleotide reductase R2 subunit
R2c: Class Ic ribonucleotide reductase R2 subunit, used here to refer to the class Ic R2 protein from *Chlamydia trachomatis*
R2lox: R2-like ligand-binding oxidase, used here to refer to the R2lox homologue 1 from *Geobacillus kaustophilus*
RNR: Ribonucleotide reductase
sMMO: Soluble methane monooxygenase
XAS: X-ray absorption spectroscopy

## Footnotes

1 The term R2lox will hereafter be used to refer to the R2lox homologue 1 from *G. kaustophilus* unless otherwise specified.
